# Tunability of Hybrid Silica Xerogels: Surface Chemistry and Porous Texture Based on the Aromatic Precursor

**DOI:** 10.3390/gels9050382

**Published:** 2023-05-05

**Authors:** Beatriz Rosales-Reina, Guillermo Cruz-Quesada, Nataly Padilla-Postigo, Marian Irigoyen-Razquin, Ester Alonso-Martínez, María Victoria López-Ramón, Maialen Espinal-Viguri, Julián J. Garrido

**Affiliations:** 1 Department of Science, Institute for Advanced Materials and Mathematics (INAMAT2), Public University of Navarre (UPNA), Campus Arrosadía, 31006 Pamplona, Spain; 2 IES Plaza de la Cruz, Calle de San Fermín, 51, 31003 Pamplona, Spain; 3 Department of Inorganic and Organic Chemistry, Faculty of Experimental Sciences, University of Jaen, 23071 Jaen, Spain

**Keywords:** xerogels, ORMOSILs, hybrid materials, tetraethoxysilane, surface chemistry, porous texture

## Abstract

The interest in new materials with specific properties has increased because they are essential for the environmental and technological needs of our society. Among them, silica hybrid xerogels have emerged as promising candidates due to their simple preparation and tunability: when they are synthesised, depending on the organic precursor and its concentration, their properties can be modulated, and thus, it is possible to prepare materials with à la carte porosity and surface chemistry. This research aims to design two new series of silica hybrid xerogels by co-condensation of tetraethoxysilane (TEOS) with triethoxy(*p*-tolyl)silane (MPhTEOS) or 1,4-bis(triethoxysilyl)benzene (Ph(TEOS)_2_ and to determine their chemical and textural properties based on a variety of characterisation techniques (FT-IR, ^29^Si NMR, X-ray diffraction and N_2_, CO_2_ and water vapour adsorption, among others). The information gathered from these techniques reveals that depending on the organic precursor and its molar percentage, materials with different porosity, hydrophilicity and local order are obtained, evidencing the easy modulation of their properties. The ultimate goal of this study is to prepare materials suitable for a variety of applications, such as adsorbents for pollutants, catalysts, films for solar cells or coatings for optic fibre sensors.

## 1. Introduction

Organic–inorganic hybrid materials continue to arouse interest today because the synergy between both components bestows them with unique properties suitable for various applications [1,2,3,4]. Among these hybrid materials, organically modified silicates, or ORMOSILs, stand out for their diversity of morphologies, porous textures and surface chemistries, making them versatile candidates for use as coatings for materials [5,6,7], and as chemical membranes for optical fibre sensors [8,9,10].

Silicon hybrid xerogels are ORMOSILs synthesised by the well-known sol-gel method; this method is simple, requires mild conditions and also allows the obtainment of materials with very different properties depending on the synthesis parameters, such as the amount of water added, the proportion of monomers, the gelling and drying temperatures, the amount of catalyst used and the pH of the synthesis media, among others [11,12]. These xerogels can be obtained by following an indirect (grafting) or direct (co-condensation) synthesis strategy [13]. The indirect method produces the hydrolysis and the consecutive condensation of one or more organosilanes (R_x_(Si(OC_2_H_5_)_4-x_) with the surface silanols of a previously synthesised silica material, resulting in the covalent anchoring of the organic groups exclusively on the surface of the xerogel. On the other hand, in the co-condensation strategy, the hydrolysis and condensation of an inorganic precursor (typically tetraethoxysilane, TEOS, or tetramethoxysilane, TMOS) with an organosilane occur at the same time, thus guaranteeing that the organic moieties are homogeneously distributed throughout the entire silica matrix. The direct method also has the advantage of allowing the design of xerogels with chemical and textural properties on demand, as the properties of the material depend on the selected organosilane and its molar ratio with respect to the pure inorganic silica precursor. This versatility is reflected in a variety of recent studies, where hybrid xerogels synthesised by this strategy have been used as coatings for diverse materials [14,15], anchors for luminescent compounds [16,17], filters for photovoltaic panels [18], matrices for compounds with biomedical applications [19,20] and adsorbents or gas separators [21,22].

Among the most widely used families of organosilanes for the preparation of hybrid xerogels, phenylsilanes stand out for providing the resulting material greater thermal stability, affinity with organic molecules and hydrophobic character than alkylic silanes [23,24,25,26,27]. This work aims to prepare and characterise two new series of hybrid xerogels bearing various molar proportions of triethoxy(*p*-tolyl)silane (MPhTEOS series) and 1,4-bis(triethoxysilyl)benzene (Ph(TEOS)_2_ series). The organic precursor MPhTEOS has previously been used in the synthesis of other organosilanes and as a coupling agent in the preparation of ORMOSILs [28,29], while Ph(TEOS)_2_ is a bridging precursor used in the synthesis of mesoporous silica [30,31,32], but to date, the synthesis of hybrid xerogels through their direct co-condensation without the use of surfactants has not been yet reported. The xerogels obtained were characterised by infrared spectroscopy (FT-IR), ^29^Si nuclear magnetic resonance (^29^Si NMR), X-ray diffraction (XRD), thermogravimetric analysis (TGA), differential scanning calorimetry (DSC), N_2_, CO_2_ and H_2_O vapour adsorption–desorption isotherms and scanning electron microscopy (SEM). Their full characterisation indicates that MPhTEOS materials become more microporous and hydrophobic as the molar percentage of the precursor increases, while those of Ph(TEOS)_2_ are more hydrophilic and mesoporous, evidencing the tunability and diversity of porous textures and the chemical properties that can be obtained with small changes in the structure and the molar proportion of the organic precursors used. The xerogels synthesised in this study could be of great interest for the preparation of chemical membranes for optic fibre sensors responsive to a wide range of analytes, as both series of materials possess very different properties, both in terms of their porosity and their affinity for polar and nonpolar molecules.

## 2. Results and Discussion

### 2.1. FT-IR

**Figure 1:**
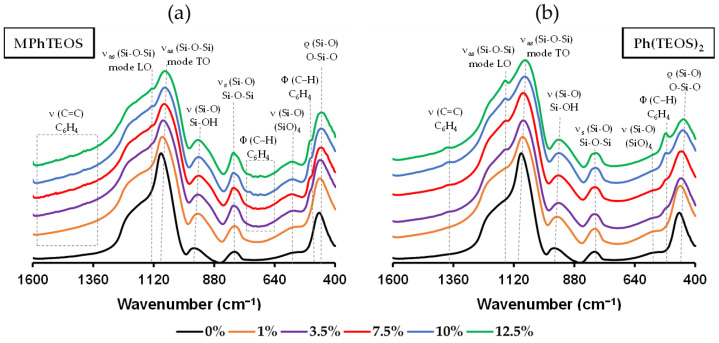
FT-IR spectra in the range of 1600–400 cm^−1^ of the reference material and the hybrid xerogels: (**a**) MPhTEOS series and (**b**) Ph(TEOS)_2_ series.

Figure 1 depicts the FT-IR spectra in the range of 1600–400 cm^−1^ of the reference material and both series of hybrid xerogels (the 4000–2750 cm^−1^ range is displayed in Figure S1 of the Supplementary Materials). The spectra present all the representative absorption bands of a silica material (the modes of vibration of Si−OH and Si−O−Si bonds). In the range of 1200–1000 cm^−1^ the band resulting from the vibrational modes of the siloxane bonds that form the different silica structures (cyclic, bicyclic, linear and branched species) is displayed [33]. However, in the spectra of both hybrid series, an emerging band is observed in this range due to the variation in the proportion of these species caused by the inclusion of the organic precursor in the network (at 1129 cm^−1^ for MPhTEOS and 1150 cm^−1^ for Ph(TEOS)_2_) [34]. Additionally, the difference in the frequency of this emerging band for each series indicates that each precursor favours the formation of different species. In fact, in the spectra of the reference and the MPhTEOS series, a shoulder at 570 cm^−1^ associated with 4-membered rings, (SiO)_4_, is observed (while it is barely visible in the Ph(TEOS)_2_ series). This is relevant because (SiO)_4_ rings are the building blocks of ordered structures, such as close/open cages and ladder-like polyhedral oligomeric silsesquioxanes (POSS) [35,36].

In addition, the bands corresponding to the benzene rings are observable in the spectra of the xerogels with the highest proportion of precursor (≥7.5%): (i) 3080–3020 cm^−1^, assigned to the stretching vibrations (ν) of C−H bonds (Figure S1); (ii) 1608–1452 cm^−1^, which belongs to the stretching vibrations of the C−C bonds; and (iii) 500 cm^−1^ due to the deformation (Φ) of the C−H bonds. All the bands and their proposed assignments are displayed in Table 1.

### 2.2. ^29^Si NMR

**Figure 2:**
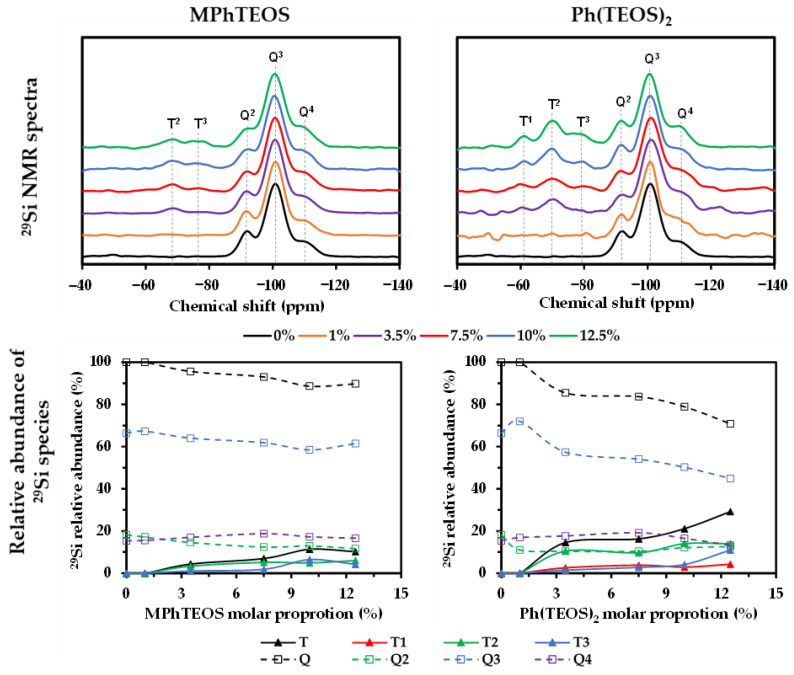
^29^Si NMR spectra and relative abundance of condensed species with respect to the molar percentage of the reference material and both series of hybrid xerogels.

To evaluate the degree of condensation and the relative abundance of silicon species in the materials, their ^29^Si nuclear magnetic resonance spectra were obtained (^29^Si NMR; the notation of silicon species is described in Section 4.3). Figure 2 depicts the spectra of both series, normalised to the signal of the most abundant species (Q^3^), and the evolution of the relative abundance of T species (T^1^ + T^2^ + T^3^) and Q species (Q^2^ + Q^3^ + Q^4^) with respect to the molar percentage of MPhTEOS and Ph(TEOS)_2_, respectively, and Table 2 displays the chemical shifts and integrals of the T signals (Q species are depicted in Table S1).

The silicon species from the hybrid precursors (T) are observed in the range of −60 to −80 ppm, and those from TEOS (Q) in the range of −80 to −120 ppm. Among the T species, the semi-condensed ones, T^2^, are the most abundant for all the materials, denoting that the complete condensation of the organic precursors is not favoured during the sol-gel process. Additionally, in the spectra of the Ph(TEOS)_2_ series, a new signal emerges at −61 ppm as the content of the precursor increases, which corresponds to the least condensed silicon species (T^1^) and has not been detected in previously studied hybrid materials [43,44]. In both series, the Q^3^/Q ratio decreases with the molar percentage of precursor, while the T^3^/T ratio increases, reaching the T^2^/T ratio at the highest molar percentages of precursor.

The percentage of the hybrid precursor does not affect significantly the chemical shifts of the T and Q species. In addition, the chemical shifts of the T species are less negative than those of the Q species (−92.1, −100.9 and −109 ppm for Q^2^, Q^3^ and Q^4^ respectively, Table S1). This can be explained by the fact that the organic substituents are less electron-withdrawing than the ethoxy groups, which is translated into fewer electropositive silicon atoms in the organic precursors than in TEOS, thus making them less prone to being affected by an external magnetic field (shielding effect) [45,46]. Comparing the chemical shifts of the materials with the same precursor percentages, the T^2^ and T^3^ signals are more negative for the Ph(TEOS)_2_ series than for the MPhTEOS series, indicating a greater positive charge density in the silicon atoms of the latter. This is relevant because the higher the positive charge in the silicon atoms, the more favoured nucleophilic attack becomes, and therefore, the greater the degree of condensation in the materials [47]. Indeed, the presence of T^1^ species in the Ph(TEOS)_2_ series indicates that these materials are less condensed than those prepared with MPhTEOS. As demonstrated by H. Saito et al., in the acidic hydrolysis of Ph(TEOS)_2_, complete hydrolysis occurs first in one of the silicon moieties bonded to the phenyl ring instead of the partial and simultaneous hydrolysis of both [48], which is translated into a kinetic limitation of condensation reactions.

The degree of condensation of a silica xerogel is intrinsically related to its internal order because the ordered structures (POSS) in the silica matrix are composed of highly condensed species such as T^3^, Q^3^ and Q^4^ [49]. Therefore, both series will tend to be amorphous, as T^2^ species are the most abundant species; however, in both series the proportion of T^3^ species increases with the molar percentage of precursor (T^3^/T in Table 2). This observation suggests that the xerogels will be amorphous up to a percentage of precursor, from which it would be possible to detect a certain degree of nanostructuration. A previous study of a series of hybrid xerogels prepared by co-condensation of TEOS and triethoxy(*p*-chlorophenyl)silane (ClPhTEOS) found that ordered domains were first detected when the ratio of T^3^/T reached 36.9% (10% molar percentage of ClPhTEOS) [43]. Therefore, based on this study, ordered domains in these materials might be expected from a 10% molar percentage of MPhTEOS (T^3^/T = 57%) and a 12.5% molar percentage of Ph(TEOS)_2_ (T^3^/T = 37.5%).

### 2.3. X-ray Diffraction Analysis

**Figure 3:**
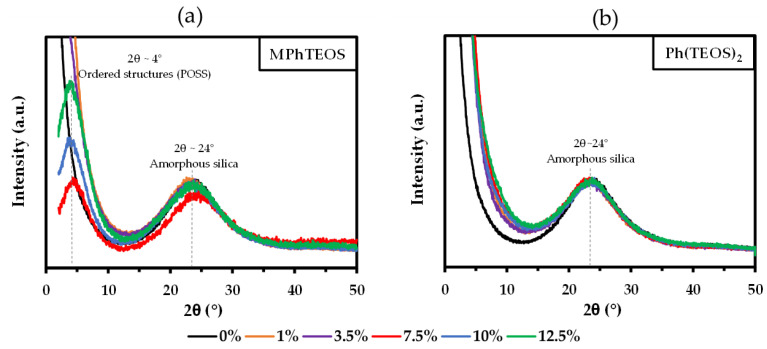
X-ray diffraction patterns of the reference material and the hybrid xerogels of: (**a**) MPhTEOS series and (**b**) Ph(TEOS)_2_ series.

Figure 3 depicts the diffraction patterns of both series of hybrid xerogels, and Table S2 in the Supplementary Materials shows the bond angles and bond distances calculated using Bragg’s law for each maximum. All the patterns are dominated by a diffraction maximum at 2θ~24°, which is characteristic of amorphous silica and associated with the distance between silicon atoms bonded by siloxane bridges [50]. Additionally, MPhTEOS xerogels with a molar percentage equal to or higher than 7.5% (Figure 3a) present a diffraction maximum at 2θ~4°, which is associated in the literature with POSS, ordered structures formed by four-folded rings, (SiO)_4_ [44,51,52,53]. This observation is consistent with the relation between the local structuration of the materials and their condensation degree (T^3^/T ratio), although for MPhTEOS xerogels local order is detected at a lower T^3^/T ratio than expected [43], and in the case of the Ph(TEOS)_2_ xerogels, ordered domains are not detected even for the materials with the highest percentages of organic precursor.

### 2.4. TGA and DSC

**Figure 4:**
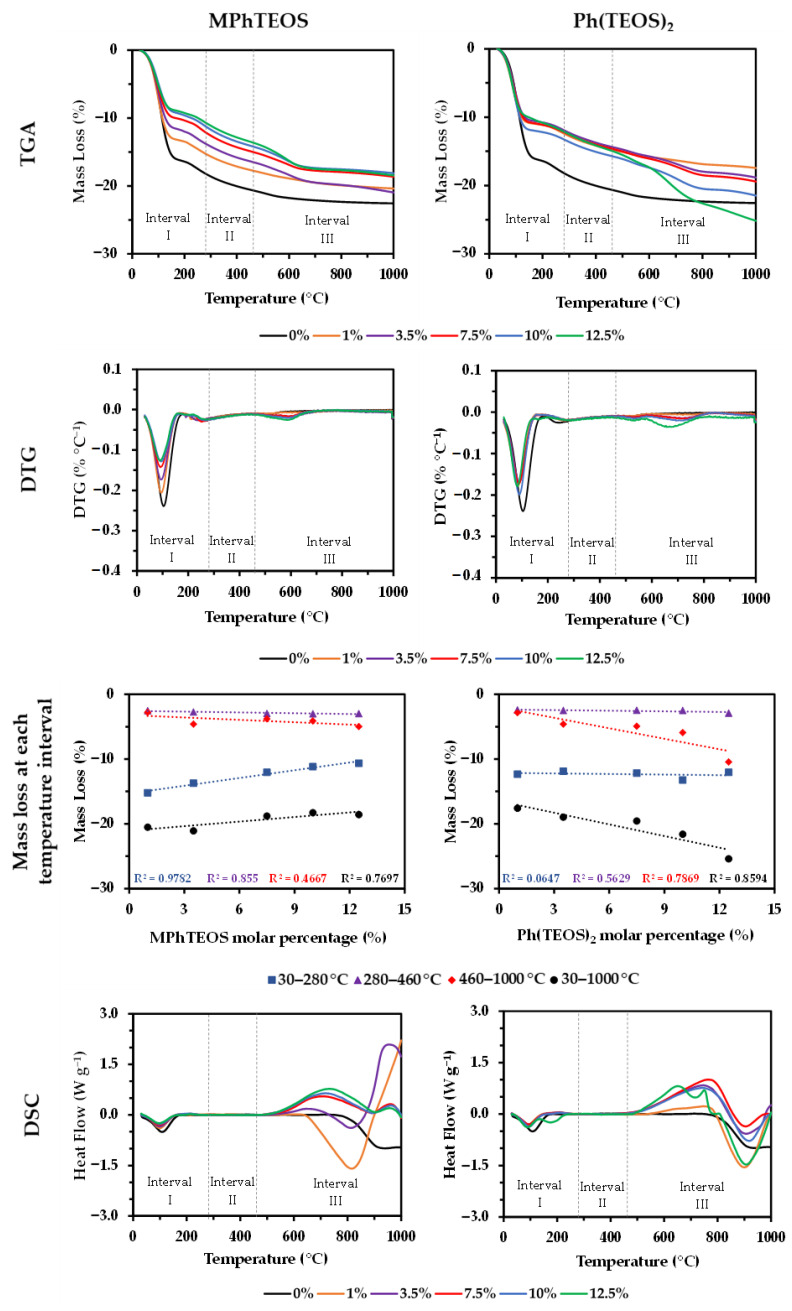
Thermograms, thermograms first derivatives, mass loss with respect to the organic precursor and DSC curves of the reference material and both series of hybrid xerogels.

Figure 4 depicts the thermograms (TG), the first derivatives of the thermograms (DTGs), the mass loss in each interval of temperatures and the differential scanning calorimetry curves (DSC curves) for all the hybrid xerogels. The TGs of the MPhTEOS series show how an increase in the molar percentage of precursor leads to a lower weight loss, while in the Ph(TEOS)_2_ series, the opposite occurs. Three degradation processes were identified in the DTGs at different temperature intervals: (i) Interval I (30–280 °C): an endothermic process associated with the elimination of physisorbed water in the pores of the materials is observed. For the MPhTEOS series, the xerogels registering the lowest mass loss are those with the highest molar percentage of precursor, as an increase in non-polar tolyl groups reduces the number of hydrophilic surface silanols. For the Ph(TEOS)_2_ series, a similar amount of water is lost in all the xerogels, and thus the greater or lesser retention of water molecules in the materials is not only due to the hydrophobic character of the precursor but also depends on the porous texture of the materials, which will be thoroughly explored in the next section by the adsorption–desorption isotherms of different adsorbates. Also noteworthy is the presence of a second endothermic process between 140 and 240 °C for 12.5Ph(TEOS)_2_. The flammability temperature of the hybrid precursor is predicted to be 150.1 ± 23.6 °C [54]; therefore this mass loss could be due to the removal of precursor molecules that are weakly anchored to the xerogel (T^1^ species). (ii) Interval II (280–460 °C): a very small loss of mass is observed for all the materials, probably due to the loss of water molecules from the condensation of the surface silanols [55,56]. (iii) Interval III (460–1000 °C): a single exothermic process is observed for the whole MPhTEOS series and up to a 10% molar percentage of precursor for the Ph(TEOS)_2_ series, while for the 12.5Ph(TEOS)_2_ xerogel, two exothermic processes take place. This is probably due to its heterogeneity because the limit of the precursor that the system can assimilate is close to being reached, as previously demonstrated for the ClPhTEOS series [43].

### 2.5. N_2_, CO_2_ and H_2_O_(v)_ Adsorption–Desorption Isotherms

**Figure 5:**
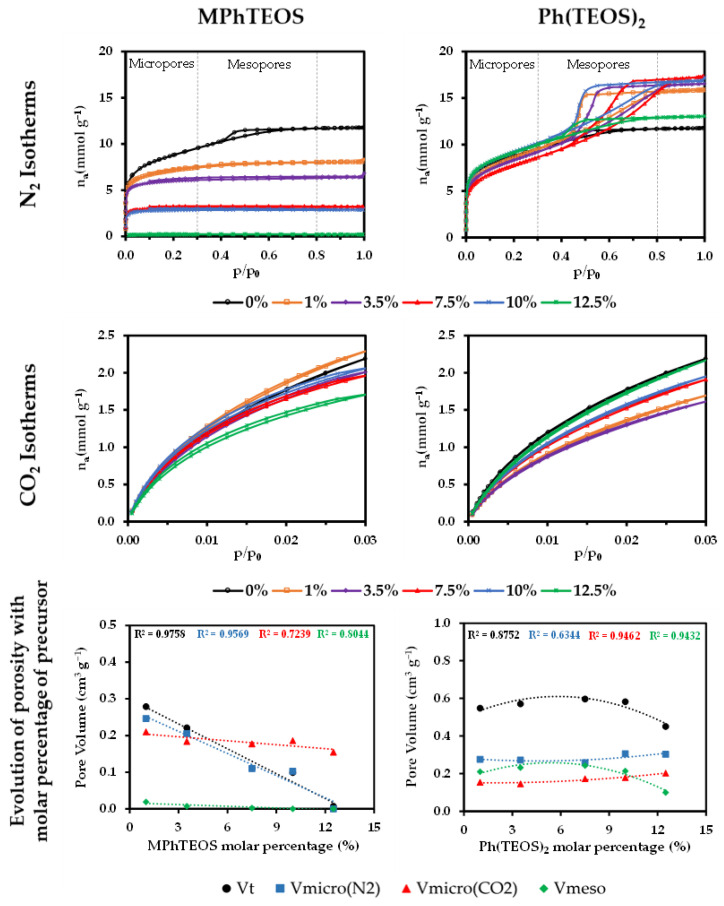
N_2_ isotherms (−196 °C), CO_2_ isotherms (0 °C) and pore volume with respect to the molar percentage of the organic precursor of the reference material and both series of hybrid xerogels.

To study the textural properties of the xerogels, N_2_ and CO_2_ isotherms were obtained. Figure 5 depicts the isotherms of the materials and the representation of the pore volume with respect to the molar percentage of organic precursor. Table 3 displays the textural parameters obtained from the data of the isotherms by applying the Brunauer–Emmett–Teller and Dubinin–Raduskevich methods (BET and DR, respectively).

The reference xerogel (in black) exhibits a mixed type I(b)–IV(a) N_2_ isotherm, which is characteristic of micro-mesoporous materials. On the other hand, the isotherms of the MPhTEOS series are characteristic of microporous materials (type I), while those of the Ph(TEOS)_2_ series are representative of mesoporous materials with a narrow pore size distribution (type IV(a) with a hysteresis loop H2(a)) [57].

**Figure 6:**
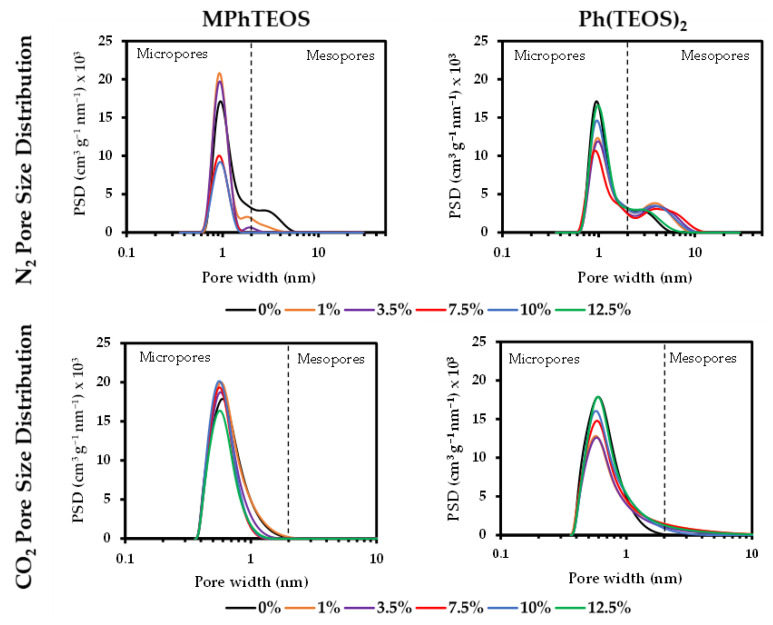
Pore size distributions (PSD) obtained from N_2_ and CO_2_ isotherms of the reference material and both series of hybrid xerogels.

As can be gathered from Figure 5, MPhTEOS xerogels adsorb less N_2_ than the reference, and the adsorption decreases as the molar percentage of MPhTEOS increases, implying that the materials become more microporous. This is reflected in a lower specific surface area as the amount of precursor rises, e.g., 1MPhTEOS has a surface area of 590 m^2^ g^−1^, while 10MPhTEOS has a surface area of 255 m^2^ g^−1^ (Table 3). 1MPhTEOS presents a type I(b) isotherm, that is, it is a microporous material with a wide distribution of micropore sizes. The 3.5MPhTEOS, 7.5MPhTEOS and 10MPhTEOS materials show a type I(a) isotherm, denoting a narrow pore distribution, and 12.5MPhTEOS barely adsorbs N_2_, which makes it impossible to know the type of isotherm and to obtain any data. It is noteworthy that this series is more microporous than the ClPhTEOS series studied previously, as for the same molar percentage of organic precursor, its materials present smaller pore volume and lower surface area [43]. Regarding CO_2_ adsorption, except for 1MPhTEOS, all the xerogels of this series adsorb less CO_2_ than the reference. However, xerogels with a molar percentage of precursor equal to or greater than 7.5% have a higher volume of micropores using CO_2_ as the adsorbate than that obtained with N_2_, consistent with their ultramicroporous nature. Indeed, their pore size distributions (PSD) calculated from N_2_ and CO_2_ isotherms indicate a pore size under 2 nm (Figure 6).

In contrast to the MPhTEOS series, Ph(TEOS)_2_ materials adsorb more N_2_ than the reference. However, the volume of mesopores and total pore volume increase with the molar percentage of organic precursor up to 7.5%, and from this value, both decrease up to a molar percentage of 12.5% (Figure 5, bottom right, second-degree polynomial adjustment). The micropore volume of the series decreases slightly up to 7.5% organic precursor, and then, from that percentage, the micropore volume increases, consistent with the higher specific surface area observed for these materials compared to the reference (697 m^2^ g^−1^ for TEOS and 744 m^2^ g^−1^ for 10Ph(TEOS)_2_, Table S2). The change in the trend at 7.5% Ph(TEOS)_2_ may be related to a higher degree of condensation favouring the formation of micropores. From the CO_2_ isotherms, it can be deduced that all the materials of this series adsorb less CO_2_ than the reference, and it is given that V_micro(N2)_ > V_micro(CO2)_, which is indicative of the predominance of micropores with a diameter larger than 0.7 nm. This statement is confirmed by the PSDs calculated for both adsorbates in this series: PSDs derived from N_2_ isotherms show how the volume of micropores centred at 1 nm increases and that of the mesopores centred at 4 nm decreases as the amount of organic precursor rises, while PSDs obtained from the CO_2_ isotherms show that most of the pores are below 2 nm in the materials with a molar percentage equal to or higher than 7.5% of Ph(TEOS)_2_ (Figure 6).

**Figure 7:**
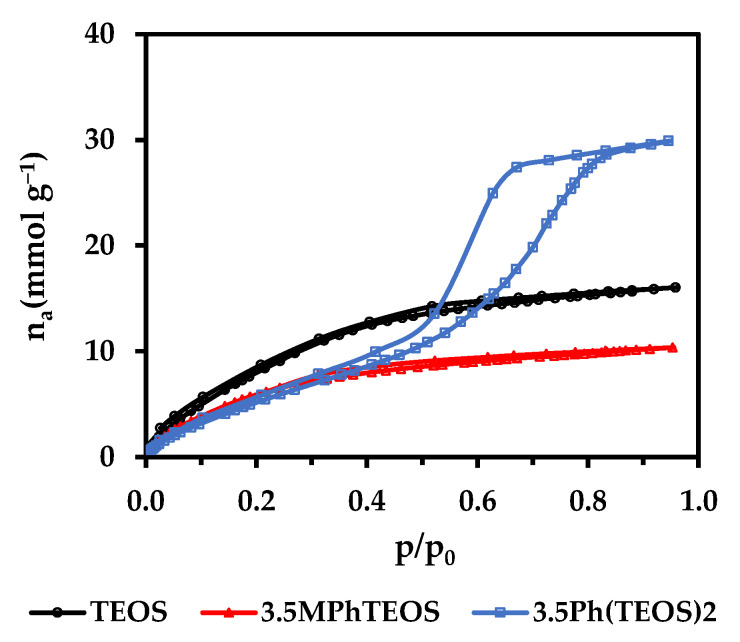
H_2_O_(v)_ adsorption–desorption isotherms (25 °C) of the reference, 3.5MPhTEOS and 3.5Ph(TEOS)_2_ xerogels.

In addition, the water retention observed in the TGAs for both series is consistent with the textural information extracted from the isotherms. The decrease of physisorbed water in the MPhTEOS xerogels as the organic precursor amount rises (Figure 4) is not only due to the hydrophobic nature of the tolyl moiety but also due to the progressive decrease of V_micro(N2)_ and, consistently, the reduction of the adsorption capacity. On the contrary, the physisorbed water of the Ph(TEOS)_2_ series remains the same for all the materials regardless of the amount of hybrid precursor, which would be related to their similar volume of micropores and mesopores (V_meso(N2)_~V_micro(N2)_). To verify this hypothesis, water vapour isotherms (H_2_O_(v)_ at 25 °C) of the reference material, 3.5MPhTEOS and 3.5Ph(TEOS)_2_ were obtained (Figure 7). These materials were chosen to compare the water isotherms of both series, avoiding the ultramicroporous nature of MPhTEOS xerogels with a higher content of organic precursor.

The data of the water isotherms displayed in Table 4 reveal that 3.5Ph(TEOS)_2_ not only adsorbs more water than the reference but also adsorbs almost 3 times more than 3.5MPhTEOS. Up to p/p_0_~0.3, both hybrid xerogels adsorb practically the same quantity of water, and less than the reference, as both have lesser volumes of micropores. In contrast, from p/p_0_ > 0.3, the adsorption of 3.5Ph(TEOS)_2_ is more pronounced than that of 3.5MPhTEOS because the mechanism of adsorption changes in the mesopores once the monolayer is completed by the physisorbed water, and capillary condensation starts to take place (the opening of the hysteresis cycle in the isotherm). Finally, from p/p_0_ > 0.6 3.5Ph(TEOS)_2_ adsorbs more water than the reference. All these observations, together with the fact that the characteristic energy of adsorption (E_c(H2O)_) is similar in the three materials, confirm that the adsorption of water molecules is dependent not only on the hydrophobic nature and the amount of organic precursor but mostly on the porous texture of the xerogels, which in turn is linked to the hybrid precursor used in their synthesis.

### 2.6. Morphological Structure

**Figure 8:**
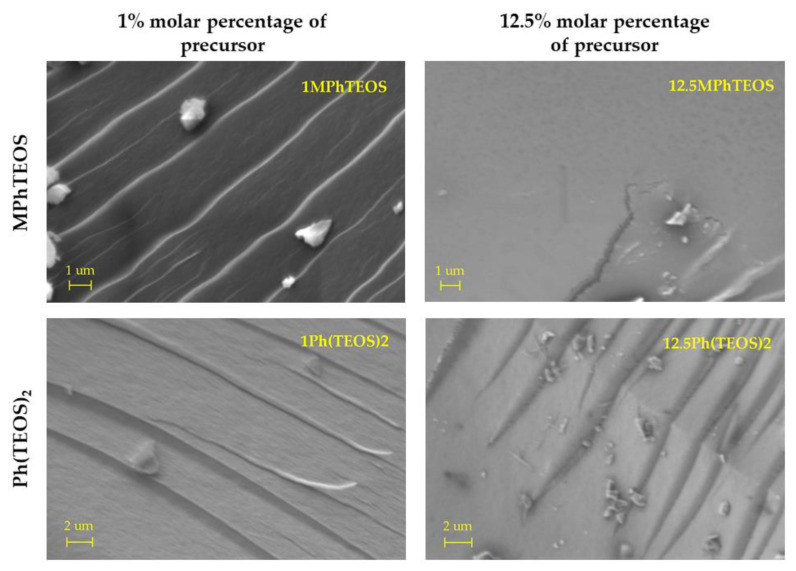
SEM micrographs of 1MPhTEOS, 12.5MPhTEOS, 1Ph(TEOS)_2_ and 12.5Ph(TEOS)_2_ materials.

To study the morphological changes on the surfaces of the hybrid xerogels, scanning electron microscopy (SEM) was performed. The micrographs of 1MPhTEOS, 12.5MPhTEOS, 1Ph(TEOS)_2_ and 12.5Ph(TEOS)_2_ are shown in Figure 8.

The 1MPhTEOS micrograph shows a morphology based on a series of superimposed layers with little roughness. On the other hand, the material with the maximum content of MPhTEOS has a smooth and fractured surface due to the tension generated during syneresis. The loss of roughness was observed before in the micrographs of the ClPhTEOS series [43], and it is related to the progressive increase of micropores and the absence of mesopores as the amount of organic precursor rises. The micrographs of 1Ph(TEOS)_2_ and 12.5Ph(TEOS)_2_ show a rough surface for both materials, and even with the highest content of organic precursor, superimposed layers are still visible, consistent with a higher volume of pores in comparison to that of 12.5MPhTEOS.

## 3. Conclusions

Two series of hybrid silica xerogels were prepared by co-condensation of tetraethoxysilane (TEOS) with two different organic precursors, triethoxy(*p*-tolyl)silane (MPhTEOS series) and 1,4-bis(triethoxysilyl)benzene (Ph(TEOS)_2_ series). A vibration band due to four-folded tetramers was observed in the FT-IR spectra of the MPhTEOS series, denoting that these xerogels might exhibit local order within the amorphous silica matrix, as (SiO)_4_ rings are the main constituents of polyhedral oligomeric silsesquioxanes (POSS). In contrast, for the Ph(TEOS)_2_ series, this band was not observed, which is consistent with the absence of internal order. ^29^Si NMR spectra of both series show that the most condensed species (T^3^, POSS building blocks) increase as the molar percentage of organic precursor rises, thus favouring local order in the materials. However, the increase in T^3^ species is less pronounced for the Ph(TEOS)_2_ series, and in addition, the lowest amounts of condensed species (T^1^) are present in these xerogels, suggesting less susceptibility to forming ordered domains. To shed light on the structuration of the materials, X-ray diffraction patterns were obtained, and a maximum associated with ordered domains was observed for the xerogels bearing the highest content of MPhTEOS, with the results being consistent with the information gathered from FT-IR and ^29^Si RMN. N_2_ and CO_2_ isotherms showed that MPhTEOS materials are microporous, and the pore volume decreases with the increase of organic precursor, whereas Ph(TEOS)_2_ materials are micro-mesoporous, a quality presumably associated with their amorphous nature and their lower degree of condensation. TGAs and water vapour isotherms revealed that Ph(TEOS)_2_ xerogels are more hydrophilic than their MPhTEOS analogues, and their hydrophilicity strongly depends on their porous nature. These two series of hybrid xerogels are an example of how, with a small variation in the organic precursor used for their preparation, it is possible to synthesise materials with very different porous textures and chemical properties. The ultimate aim of this study is the use of these materials as chemical membranes for fibre optic sensors, and to obtain coatings that are sensitive to and selective for a wide range of analytes (based on their size and polarity), the control of their textural and chemical properties is of great relevance.

## 4. Materials and Methods

### 4.1. Materials

The siliceous precursors TEOS (tetraethoxysilane, purity > 99%), MPhTEOS ((4-methylphenyl)triethoxysilane, purity > 95%) and Ph(TEOS)_2_ ((4-(triethoxysilyl)phenyl)silane, purity > 95%) were purchased from Sigma-Aldrich (St. Louis, MO, USA). Absolute ethanol (Emsure ^®^) and hydrochloric acid (HCl, 37% w/w) were supplied by Merck (Darmstadt, Alemania), and potassium bromide (grade FT-IR) was supplied by Sigma-Aldrich (St. Louis, MO, USA). All chemicals were used as received without further purification.

### 4.2. Synthesis of Hybrid Silica Xerogels

The synthesis of hybrid xerogels was carried out according to the procedure described in a previous work [44], where the molar ratio of (TEOS + RTEOS):ethanol:water was set at 1:4.75:5.5 for all series and where the amounts of reagents and solvents were adjusted to obtain 20 mL of alcogel. The xerogels were named according to the organic precursor (RTEOS) and its molar percentage (e.g., 10MPhTEOS and 10Ph(TEOS)_2_ for the xerogels with a molar percentage of 10% of MPhTEOS and Ph(TEOS)_2_ respectively). In addition to both series of hybrid xerogels, a reference material synthesised only with TEOS was prepared.

**Figure 9:**
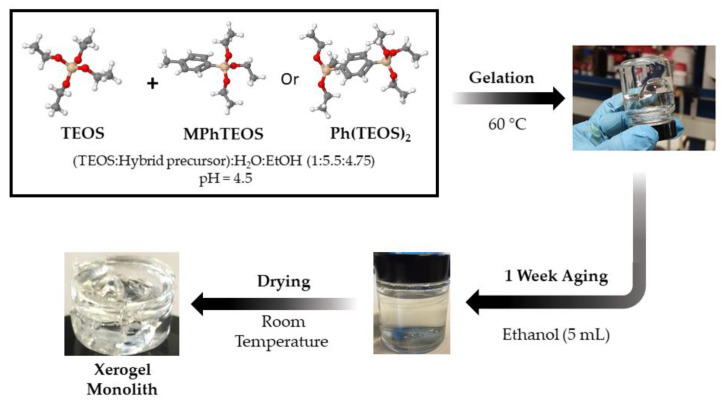
Synthesis scheme of the reference material and both series of hybrid xerogels.

Initially, for the synthesis of the hybrid xerogels, TEOS was first mixed with the corresponding organic precursor in a 30 mL container (ϕ 2.5 cm, screw-on plastic lid, Fischer Scientific Ltd., Madrid, Spain). Absolute ethanol was then added, followed by dropwise addition of Milli-Q-grade water with magnetic stirring to facilitate miscibility, maintaining, in all cases, molar ratios of (TEOS:RTEOS):ethanol:water at 1:4.75:5.5. Subsequently, hydrochloric acid solution (0.05 M) was dosed into the alcogel using an automatic buret (Tritino mod. 702 SM, Metrohm, Herisau, Switzerland) until the pH reached 4.5. The closed containers were placed in a thermostatically controlled oven at 60 °C (J.P. Selecta S.A, Barcelona, Spain) until gelling (the time at which the shape of the materials does not change when the container is tilted). Subsequently, 5 mL of ethanol was added to cure the alcogel at room temperature for 1 week. The recipients were then opened to dry the materials, and they were then covered with Parafilm™, which was perforated to facilitate solvent evaporation. The monoliths were considered dry when no significant change in their mass was observed. The scheme of the synthesis is displayed in Figure 9.

### 4.3. Characterisation of Hybrid Silica Xerogels

The amorphous nature of the silica xerogels requires numerous techniques to obtain information about their structure and properties. To use these techniques, material samples were first crushed in a mortar and then placed in a vacuum thermostatic desiccator (Vacuo-Temp, JP. SELECTA) at 100 °C to remove any moisture.

FT-IR spectra were recorded using 25 scans and a resolution of 4 cm^−1^ in a Jasco spectrometer (mod. 4700, Japan). Spectra were obtained from KBr tablets (200 mg) with different amounts of dispersed sample: (i) 2 mg, to obtain information on the –OH groups and phenyl C–H bonds in the range of 4000–2200 cm^−1^, and (ii) 0.6 mg, to avoid saturation of the Si–O–Si asymmetric stretching signal in the range of 2200–400 cm^−1^ [58].

^29^Si Cross-polarization magic-angle spinning (CP MAS) solid-state NMR spectra were recorded on a Bruker AV-400 MHz spectrometer (Billerica, MA, USA) using TMS as the reference and operating at 79.5 MHz and 5 kHz. The spectra were ^1^H-decoupled, and 800 scans were acquired per spectrum. The classical notation for these types of materials was used to assign the different signals: *T* for the signals derived from the organic precursor silicon atoms and *Q* for the ones from the inorganic precursor TEOS. Depending on the number of siloxane bridges in each silicon atom, a superscript i was added (T^i^ and Q^i^) [46].

A PANalytical Empyrean XRD instrument (Empyrean, Almelo, The Netherlands) provided with a copper anode and a graphite monochromator was used for the obtention of the X-ray diffraction patterns, and the measurements were performed in the range of 2 ≤ 2θ ≤ 50°.

Simultaneous thermogravimetry and differential scanning calorimetry (TGA-DSC) analysis of the materials were performed using a TGA/DSC 3+ series thermogravimetric analyser (Mettler Toledo, Greifensee, Switzerland) under a constant N_2_ flow of 50 mL min^−1^. Samples (~20 mg) were placed in 70 µL alumina crucibles and heated from 30 to 1000 °C at a rate of 10 °C min^−1^. An experiment under the same conditions and without a sample was carried out and used as a blank.

N_2_ (−196 °C) and CO_2_ (0 °C) adsorption–desorption isotherms were obtained using a volumetric adsorption system (ASAP2020, Micromeritics, Norcross, GA, USA). First, 150 mg of sample was used for each isotherm, placed into a Pyrex glass tube, and degassed at 150 °C up to a residual vacuum of less than 0.66 Pa. For N_2_ analysis the tube was coated with a Teflon isothermal jacket and immersed in a Dewar flask containing liquid nitrogen, whereas for CO_2_ analysis the tube was coated with a metal isothermal jacket and immersed in a thermostatised recirculation bath using a 50% water/ethylene glycol mixture as the refrigerant. The data were analysed using the Microactive software (version 4.06) and applying different models. The specific surface areas were calculated with Brunauer–Emmett–Teller (BET) model and the Rouquerol criteria of the data obtained with N_2_ as the adsorbate (a_BET_) [59], and the Dubinin–Radushkevich (DR) method was applied to the CO_2_ data (a_DR_) [60]. Densities (0.808 g cm^−3^ for N_2_ and 1.023 g cm^−3^ for CO_2_) were used to calculate the volume of micropores (V_micro(N2)_ + V_micro(CO2)_, calculated with the DR method, diameter (ϕ) ≤ 2 nm), mesopores (V_meso_, calculated by the subtraction of the adsorbed N_2_ at p/p◦ = 0.3 and that adsorbed at p/p◦ = 0.8, 2 < ϕ ≤ 50 nm) and total volume of pores (V_t_, considering the adsorbed N_2_ at p/p◦ = 0.95) [61].

Pore size distributions (PSD) were determined according to density-functional theory (DFT) using the SAIEUS method and software [62], and applying the “carbon-N2-77, 2D-NLDFT heterogeneous Surface” model for N_2_ adsorption and the “carbon-CO2-273, 2D-NLDFT Het Surface, pp max = 10 atm” model for CO_2_ adsorption.

H_2_O vapour adsorption isotherms were obtained at 25 °C using the same equipment and sample holder as those used for N_2_ and CO_2_ isotherms. The water vapour was dosed using the vapour kit of the equipment, and the analysis temperature was achieved using the metal isothermal jacked and the thermosthatised recirculation bath used for the CO_2_ isotherm acquisitions. The time needed for the analysis ranged from 38 to 72 h. The BET and DR models were used to determine the a_BET_ and E_c_, respectively. The liquid density of H_2_O was obtained from the literature: 0.997 g.cm^−3^ at 25 °C [63].

Micrographs were acquired using a Zeiss Model EVO 15 (Oberkochen, Germany) scanning electron microscope (SEM) at 200 kV. Samples were metallised with gold–palladium for 1 min at 10 mA.

## Figures and Tables

**Table 1:** List of FT-IR bands observed in the spectra of both series of hybrid xerogels and proposed assignation based on the literature.

Wavenumber	Vibration	Structural	Xerogel Series	References
(cm^−1^)	Assignation	Unit		
3660	ν (OH−H)	SiO−H	TEOS, MPhTEOS and Ph(TEOS)_2_	[34,37]
3450	ν (OH−H)	SiO−H (H−Bridge)	TEOS, MPhTEOS and Ph(TEOS)_2_	[34,37]
3082–3020	ν (C−H)	C_6_H_4_	MPhTEOS and Ph(TEOS)_2_	[38]
2980–2900	ν (C−H)	CH_3_	MPhTEOS	[38]
1608–1452	ν (C=C)	C=C−H	MPhTEOS and Ph(TEOS)_2_	[38]
1155	ν_as_ (Si−O−Si mode LO)	≡Si−O−Si≡	Ph(TEOS)_2_	[35,39,40]
1126	ν_as_ (Si−O−Si mode LO)	≡Si−O−Si≡	MPhTEOS	[35,39]
1090	ν_as_ (Si−O−Si mode TO)	≡Si−O−Si≡	TEOS, MPhTEOS and Ph(TEOS)_2_	[35,39,41]
955	ν (Si−O)	≡Si−O−H	TEOS, MPhTEOS and Ph(TEOS)_2_	[35,40]
800	ν_s_ (Si−O)	≡Si−O−Si≡	TEOS, MPhTEOS and Ph(TEOS)_2_	[38,41]
800 (incipient band)	T_δ,y_ C−H	C_6_H_4_	MPhTEOS	[38]
735–696	Φ C−H	C_6_H_4_	MPhTEOS	[38]
570	ν Si−O	Si−O_2_ (SiO)_4_	MPhTEOS and Ph(TEOS)_2_	[42]
515	Φ C–H	C_6_H_4_	Ph(TEOS)_2_	[38]
498	Φ C–H	C_6_H_4_	MPhTEOS	[38]
455	ρ Si−O	O−Si−O	TEOS, MPhTEOS and Ph(TEOS)_2_	[34,41]

ν, Stretching vibration; ν_s_, Symmetric stretching vibration; ν_as_, Asymmetric stretching vibration; Τ_δ,y_ C−H, wagging out and inside the plane; Φ, deformation out and inside the plane; ρ, rocking; LO, Longitudinal optical vibration mode; TO, Transversal optical vibration mode; (SiO)_4_, 4-fold ring.

**Table 2:** Chemical shifts and integral areas of the T signals of both series of hybrid xerogels.

Precursor	Molar Percentage (%)	^29^Si NMR (ppm)			Band Areas ^b^				T^3^/T
		T^1^	T^2^	T^3^	T	T^1^	T^2^	T^3^	(%)
MPhTEOS	1	a	a	a	a	a	a	a	c
	3.5	a	−68.7	−77.0	4.4	a	3.3	1.0	23.5
	7.5	a	−68.3	−76.5	7.0	a	5.1	1.8	26.4
	10	a	−68.0	−75.8	11.3	a	4.9	6.5	57.0
	12.5	a	−68.3	−77.0	10.2	a	6.0	4.2	41.0
Ph(TEOS)_2_	1	a	a	a	a	a	a	a	c
	3.5	−60.6	−70.2	−77.9	14.5	2.6	10.5	1.4	9.9
	7.5	−59.9	−70.0	−80.1	16.3	3.8	9.7	2.8	17.1
	10	−61.0	−69.7	−78.8	21.1	2.9	14.1	4.2	19.7
	12.5	−61.1	−69.7	−78.1	29.2	4.3	14.0	10.9	37.5

a, Non-detected; ^b^ T + Q = 100, Q band areas in Table S1; c, Non-calculated.

**Table 3:** Textural parameters of the reference material and both series of hybrid xerogels.

Precursor	Molar Percentage (%)	a_BET_	a_DR_	V_micro_	V_micro_	V_meso_	V_total_	E_c_ ^a^	E_c_ ^a^
		(N_2_)	(CO_2_)	(N_2_)	(CO_2_)	(N_2_)	(N_2_)	(N_2_)	(CO_2_)
		(m^2^ · g^−1^)		(cm^3^ · g^−1^)				(kJ · mol^−1^)	
TEOS	0	697	510	0.28	0.20	0.07	0.41	15.27	19.71
MPhTEOS	1	590	500	0.25	0.21	0.02	0.28	16.39	19.54
	3.5	506	438	0.21	0.18	0.01	0.22	19.46	19.76
	7.5	276	423	0.11	0.18	d	0.11	19.35	20.15
	10	255	443	0.10	0.19	d	0.10	18.60	20.18
	12.5	b	368	c	0.16	c	c	b	20.06
Ph(TEOS)_2_	1	683	368	0.28	0.15	0.21	0.5	14.04	19.36
	3.5	666	347	0.27	0.15	0.23	0.57	13.87	19.55
	7.5	627	411	0.26	0.17	0.24	0.60	13.22	19.49
	10	744	426	0.31	0.18	0.21	0.58	13.80	19.39
	12.5	740	483	0.30	0.20	0.10	0.45	14.54	19.09

^a^ Characteristic energy from Dubinin-raduskevich; b Cannot be calculated; c The samples did not adsorb N_2_; d Pore volume lower than 0.01 cm^3^ ∙ g^−1^.

**Table 4:** H_2_O_(v)_ adsorption parameters of the reference, 3.5MPhTEOS and 3.5Ph(TEOS)_2_ materials.

Xerogel	a_BET_	V_micro_^a^	V_meso_^b^	V_total_^c^	E_c_^d^
	m^2^ · g^−1^	cm^3^ · g^−1^			kJ · mol^−1^
**TEOS**	589	0.14	0.08	0.29	7.63
3.5MPhTEOS	444	0.11	0.04	0.19	7.92
3.5Ph(TEOS)_2_	365	0.09	0.37	0.54	7.83

^a^ Micropore volume obtained from DR; ^b^ Mesopore volume obtained from isotherms (0.8 < p/p_0_ < 0.95); ^c^ Total pore volume obtained from isotherms at p/p_0_ = 0.95; ^d^ Characteristic energy from DR.

## Data Availability

The data presented in this study are available on request from the corresponding author.

## Supplementary Materials

The following supporting information can be downloaded at: https://www.mdpi.com/article/10.3390/gels9050382/s1, Figure S1: FT-IR spectra (range 4000–2750 cm^−1^) of the reference material and the hybrid xerogels of: (a) MPhTEOS series and (b) Ph(TEOS)_2_ series; Table S1: Chemical shifts and integral areas of the Q signals in the ^29^Si NMR spectra of both series of hybrid xerogels; Table S2. Bragg angles (2θ) and bond distances (d_1_ and d_2_ (nm)) calculated from XRD maxima of both series of hybrid xerogels.
