# Supplementary material for: Tunability of Hybrid Silica Xerogels: Surface Chemistry and Porous Texture Based on the Aromatic Precursor
Source: Gels. 2023 May 5;9(5):382. doi: 10.3390/gels9050382 (PMC10217688; doi:10.3390/gels9050382)
Supplement: Supplementary file 1 [file gels-09-00382-s001.zip › gels-2356822-supplementary.pdf]

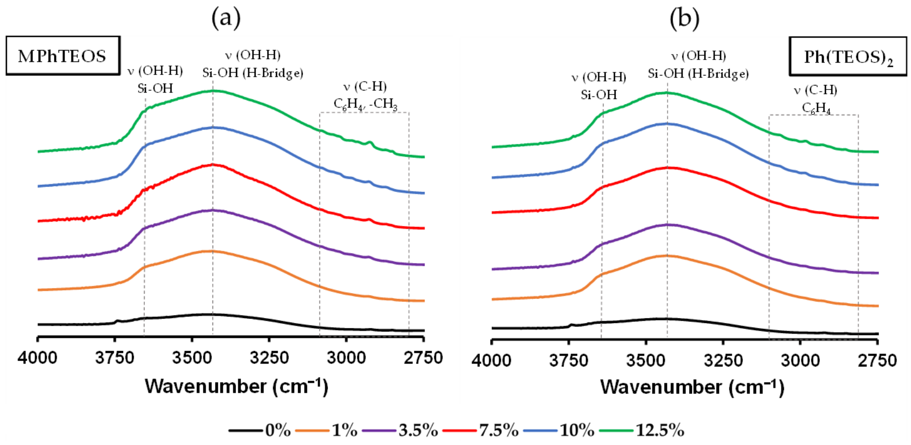

Figure S1. FT-IR spectra (range 4000–2750 cm^{-1}) of the reference material and the hybrid xerogels of: (**a**) MPhTEOS series and (**b**) $Ph(TEOS)_2$ series.

Table S1. Chemical shifts and integral areas of the Q signals in the ^{29}Si NMR spectra of both series of hybrid xerogels.

Precursor	Molar Percentage (%)	^{29}Si NMR (ppm)			Band Area			
		Q^2	Q^3	Q^4	Q	Q^2	Q^3	Q^4
TEOS	0	−91.8	−100.8	−110.0	100.0	18.2	66.4	15.4
MPhTEOS	1	−91.7	−100.8	−110.0	100.0	17.2	67.3	15.5
	3.5	−91.7	−100.7	−110.0	95.6	14.6	64.0	17.0
	7.5	−91.8	−100.8	−110.0	93.0	12.4	61.9	18.7
	10	−91.6	−100.5	−109.6	88.7	12.9	58.5	17.3
	12.5	−91.8	−100.7	−109.7	89.8	11.7	61.5	16.6
$Ph(TEOS)_2$	1	−89.8	−98.7	−108.0	100.0	11.1	72.0	16.9
	3.5	−91.6	−101.1	−110.5	85.5	10.4	57.4	17.7
	7.5	−91.5	−101.1	−110.8	83.7	10.5	54.1	19.2
	10	−91.7	−100.8	−110.0	78.9	12.1	50.2	16.5
	12.5	−91.5	−100.6	−109.8	70.8	12.6	45.0	13.2

Table S2. Bragg angles (2θ) and bond distances (d_1 and d_2 (nm)) calculated from XRD maxima of both series of hybrid xerogels.

Precursor	Molar Percentage (%)	Peak 2 θ < 10°		Peak 10° > 2 θ < 30°	
		$2θ_1$ (°)	d_1 (nm)	$2θ_2$ (°)	d_2 (nm)
TEOS	0	a	a	23.48	0.38
MPhTEOS	1	a	a	23.35	0.38
	3.5	a	a	23.82	0.37
	7.5	4.30	2.06	24.61	0.36
	10	4.43	1.99	23.78	0.37
	12.5	3.75	2.35	23.13	0.38
$Ph(TEOS)_2$	1	a	a	24.16	0.37
	3.5	a	a	23.23	0.38
	7.5	a	a	23.28	0.38
	10	a	a	24.14	0.37
	12.5	a	a	23.80	0.37

a, Non-detected.
